# Prognostic significance of morphology markers of chromosomal instability in acute leukaemia and myelodysplastic syndrome

**DOI:** 10.4314/gmj.v58i2.6

**Published:** 2024-06

**Authors:** Anju Khairwa, Mrinalini Kotru, Pooja Dewan, Shiva Narang

**Affiliations:** 1 Departments of Pathology, University College of Medical Sciences & GTB hospital, Delhi- 110095; 2 Departments of Pediatrics, University College of Medical Sciences & GTB hospital, Delhi- 110095; 3 Department of Medicine, University College of Medical Sciences & GTB hospital, Delhi- 110095

**Keywords:** Chromosomal instability, Morphology markers, Prognostic outcomes, Acute leukaemia, MDS

## Abstract

**Objective:**

This study aimed to assess the prognostic significance of various morphological markers of chromosomal instability (CI).

**Design:**

This is a cross-sectional analytical study.

**Setting:**

Single centre study from the Department of Pathology of a tertiary care centre in India.

**Participants:**

The study included samples of bone marrow aspirates (BMA) and biopsies of patients with acute leukaemia (AL) and myelodysplastic syndrome (MDS) performed between June 2019 and June 2021. Inadequate samples were excluded. We included 178 samples from 80 cases.

**Interventions:**

BMA and biopsies slides examined for CI markers like chromatin bridges, multipolar mitosis, nuclear budding, micronuclei, laggards, chromatin string (CS) and nuclear heterogeneity (NH). CI markers were correlated with the type, severity and prognosis of acute leukaemia and MDS.

**Main outcome measures:**

Evaluation of CI markers as prognostic markers in AL and MDS.

**Results:**

We included B-cell ALL (35), AML (11), MDS (04), relapse of AL (12), and remission of AL (116). All CI markers were significantly increased in AL and MDS compared to the remission group. All CI markers were significantly higher in non-responders to therapy than in responders. In regression analysis, the median (IQR) values of CS and NH were significantly higher among non-survivors than survivors.

**Conclusion:**

CI markers of morphology are significantly associated with poor prognosis, including Non-survival of the disease. These markers are easy to identify and cost-effective. We recommend incorporating morphological markers of CI in routine reporting of haematological malignancies to assist in prognostication before reports from sophisticated techniques are available.

**Funding:**

None declared

## Introduction

Chromosomal instability (CI), or genetic instability, is a persistently high rate of loss/gain of whole or part of chromosomes and is an important factor in carcinogenesis.^1^ Defects in chromosomes can induce morphological and genetic abnormalities like C spindle assembly checkpoint cohesion, etc., which may lead to malignancies.^1^ Structural alteration in chromosome size, aneuploidy, deletion, addition, and loss of heterozygosity can lead to changes in gene expression or a change in gene structure such that the protein sequence is altered.^1^ These genetic changes can either enhance or decrease the activity of proteins or change the function of newly formed proteins.

Genetic instability, like gradual accumulation of mutations, shortening of telomeres and changes in the microenvironment, are likely to enhance the possibility of malignancies.^2^,^3^

There are various techniques for CI detection, including cytokinesis block, immunohistochemistry with anti-centromeres antibodies, telomeres, and DNA doublestranded breaks, advanced conventional cytogenetics, time-lapse and fluorescent microscopy, flow cytometry, and molecular cytogenetics like fluorescent in situ hybridisation and comparative genomic hybridisation.^4^ The molecular methods are not routinely used in resourcepoor settings due to their high cost, non-availability, and lack of expertise. for performing and interpreting the method. Therefore, the present study emphasises the importance of identifying the morphological markers of CI, such as chromatin bridges, multipolar mitosis, nuclear budding, micronuclei, laggards, chromatin string, and nuclear heterogeneity, for diagnosis and prognosis. These CI markers have also been demonstrated in research cell lines and have been studied in solid tumours, but there are few studies on CI markers in haematologic malignancies.^3^,^5^ Primary cultures of solid tumours have confirmed that the abnormalities mentioned above in cancer may represent an underlying genetic instability.^5^ The role of many morphological chromosomal instability markers, as described above, used in the diagnosis and prognosis of various malignancies has been reviewed, and their inclusion in the day-to-day reporting of cancers has been discussed.^6^ The CI in acute leukaemia (AL) and myeloid dysplastic syndrome (MDS) is important, but studies related to morphologic markers of CI are very few in the literature for AL and MDS.^7^ Further, morphologic markers of CI may also be used to correlate with immunophenotypes/cytogenetics, classify the types of acute leukaemia and MDS, and assess the behaviour/aggressiveness/prognostic significance of acute leukaemia and MDS.

This study was conducted to identify and assess the prognostic significance of the various morphological markers of chromosomal instability (CI), including chromatin bridges, multipolar mitosis, nuclear budding, micronuclei, laggards, chromatin string and nuclear heterogeneity in bone marrow aspirates and biopsies of acute leukaemia and MDS cases. This is premised on our earlier pilot study.^8^

## Methods

**Figure 1:**
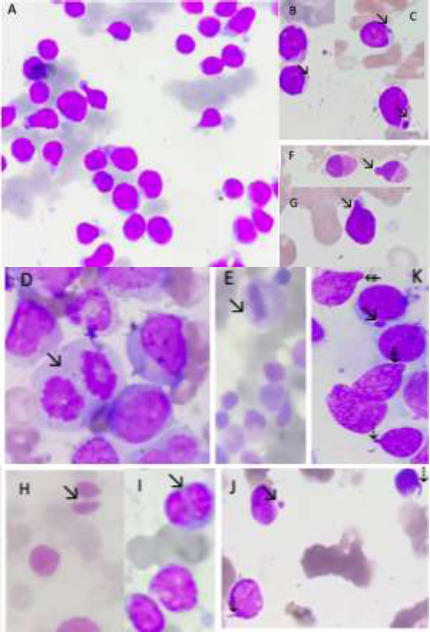
Microphotographs of bone marrow aspirate showing the distinctive features of the morphological markers of chromosomal instability (A) Nuclear heterogeneity (NH); (B and C) Micronucleus; (D) Multipolar mitosis; (E) Laggards; (F and G) Nuclear bud (NB); (H and I) Chromatin Bridge (CB; (J) Chromatin string (CS-dotted arrow) and micronucleus; (K) Multiple chromosomal instability markers seen in single oil immersion field ((NB- the arrow with two lines on a shaft, CS-dotted arrow, Micronucleus-simple arrow) Wright's (Leishman) stain; 1000x for all figures)

This retrospective cross-sectional and analytic study was performed at the University College of Medical Sciences and Guru Teg Bahadur (GTB) Hospital, Delhi, India. Bone marrow aspirate (BMA) and biopsy slides, and the clinical and laboratory data of patients diagnosed with acute leukaemia and MDS by bone marrow aspiration, biopsy, flow cytometry, and cytogenetics were retrieved and analysed between June 2019 and June 2021. Morphological details of various CI markers on bone marrow aspirate smears stained with Wright's (Leishman) stain and Haematoxylin and Eosin (H&E)-stained sections of the biopsies were examined under light microscopy.^8^ Cases used in the pilot study were excluded from this study. One BMA smear and one biopsy section were examined for CI markers for each case. Both (BMA smear and biopsy) were examined to provide average numbers of CI markers considered as standard for this study. All CI markers of morphology [micronucleus (MN), multipolar mitosis (MPM), nuclear buds (NB), chromatin bridges (CB), nuclear heterogeneity (NH), laggards (L), and chromatin string (CS)] are depicted in Figure 1 (A-K).

In this study, we determined the correlation among various morphologic markers of CI with acute leukaemia and MDS severity using univariate and multivariate analysis. The different types of AL (B/T-ALL, AML), relapse groups of disease and MDS were compared to find out the type of AL, relapsed AL and MDS associated with high scores of CI markers.

The average scores of CI markers were correlated with outcomes of acute leukaemia (ALL vs AML) and MDS patients. The clinical manifestations and response to therapy of AL and MDS patients were also correlated with survivors and non-survivors, along with CI markers.

All the morphology makers of CI were scored for each BMA and biopsy in every case. We compared CI markers in five groups: 1-ALL, 2-AML (acute myeloid leukaemia), 3-MDS, 4-Relapse cases of Acute leukaemia and 5-Remission cases of acute leukaemia. We then subgrouped these patients into three groups: group 1-patients with active disease (AL and MDS), group 2- patients with relapse of acute leukaemia and group 3- patients with the disease in remission (remission group) and compared morphology CI markers among them.

The average values of CI markers were correlated with outcomes of acute leukaemia and MDS patients. The outcomes were studied in two groups: Survivors and nonsurvivors. Both (survivors and non-survivors) must have taken the same chemotherapy regime. The endpoint for survival comparison was two years of survival probability. We also compared CI markers among survivors and non-survivors for ALL and AML. The clinical manifestations of AL and MDS patients were also compared among survivors and non-survivors. CI markers were also compared between the responder group (complete and partial response to therapy) and the non-responder group (not responding to therapy- refractory from the first cycle of treatment and relapse cases- an initial response to therapy later reoccurs) to see a therapeutic response. All patients who are follow-up lost were excluded from the analysis of the responder and non-responder group.

### Data analysis

The data obtained were analysed using STATA 14 software. The Kruskal-Wallis test for skewed variables was used to compare CI markers among various groups and Dunn's test to determine the difference between specific pair-wise groups when the Kruskal-Wallis test reported a statistically significant difference between multiple groups. We compared the clinical features and average values of the CI markers between survivors and non-survivors using the Wilcoxon rank-sum (Mann-Whitney) test (for skewed continuous variable) and the chi-square test (for dichotomous variable). Univariate linear regression analysis was used to determine the effect of variables (clinical features and CI markers of morphology) on patient outcomes (non-survival and survival). A p-value of <0.05 was considered statistically significant.

### Ethical considerations

This study was approved by the Institute's Ethics Committee, with approval number IECHR-2021-51-15. We maintained the confidentiality of data by restricting data access to limited persons. The ethics committee waived consent.

## Results

A total of 178 specimens of bone marrow aspirate and trephine biopsy samples from 80 patients (70 children, 168 samples and 10 adults, 10 samples) diagnosed with acute leukaemia or MDS were included in the study. All ten adult patients were referred to other centres and were lost to follow-up. Among the paediatric patients, 38 were deceased, 7 were on maintenance therapy, four were lost to follow-up, two patients with B-ALL were in relapse on treatment, and 29 patients were alive and in complete remission till one year of follow-up.

The median (interquartile range [IQR]) age of affected patients was 6 (4.5, 8) years, and 80% (64/80) were male. The most common clinical presentation and haematological details of acute leukaemia and MDS patients are shown in Table 1.

**Table 1:** Clinical and haematological details of acute leukaemia and MDS patients (n= 80)

SN.	Clinical features	Number(%)
**1.**	Anaemia	76(95.0%)
**2.**	Weight loss	57(71.3%)
**3.**	Loss of appetite	52(65.0%)
**4.**	Fatigue/weakness	65(81.3%)
**5.**	Fever	42(52.5%)
**6.**	Bone Pain	31(38.8%)
**7.**	Failure to thrive	31(38.8%)
**Haematological profile**		
**1.**	High total leukocyte counts (TLC) ≥ 50,000×10^6^µ/dl	40(50.0%)
**2.**	TLC >2000 - <50,000×10^6^µ/dl	39 (49.0%)
**3.**	Leukopenia (low TLC) ≤ 2000×10^6^µ/dl	1(0.8%)
**3.**	Peripheral smear blasts ranging from 0 to 97%	80(100%)
**4.**	Bone marrow aspirate (BMA) blasts ranging from 0 to 99%	80(100%)

The final diagnoses, based on BMA and Flow Cytometry (FC) findings, of the included samples were B/T-ALL (B/T-cell lymphoblastic lymphoma) (n=35), AML (n=11), MDS (n=04), relapse cases of AL (n=12), and samples of remission cases of AL (n=116). The median (IQR) scores of morphological markers of CI were as follows: micronucleus =0 (0-2), nuclear buds (NB) =2 (012), multipolar mitosis (MPM) = 0 (0-2), chromatin bridges (CB) = 0 (0-0), laggards = 0 (0-0), and chromatin String (CS) = 0 (0-3). Nuclear heterogeneity (NH) was detected in 24.2% of samples.

The average number of all CI markers on smear and section was significantly increased in AML, B-ALL, MDS and relapse cases compared to remission cases with a *P*<.0001 (Table 2).

**Table 2:** CI markers of morphology in acute leukaemia and MDS (myelodysplastic syndrome) patients (n=178 sample) at the University College of Medical Sciences and GTB Hospital, Delhi, North India, between June 2019 to June 2021

CI markers		Groups				
	B/T-ALL (n) (35)	AML (n) (11)	MDS (n) (04)	Relapse (n) (12)	Remission (n) (116)	P-value**
MN*	3(2, 5)	2(0, 8)	3.5(2, 8.5)	3(1, 6)	0(0, 0)	<0.001
NB*	21(12, 33)	15(6, 23)	6(2.6, 9)	29(20, 36)	1(0, 2)	<0.001
MPM*	3(1,6)	1(0, 3)	2(1, 3)	5(3, 6)	0(0, 0)	<0.001
CB*	1(0, 1)	0(0, 1)	0(0, 0.5)	0(0, 1)	0(0, 0)	<0.001
Laggards*	0(0,1)	0(0, 1)	0(0, 2)	0(0, 1)	0(0, 0)	<0.001
CS*	6(3, 13)	7(1, 12)	1.5(0.5, 4)	9(6, 12)	0(0, 0)	<0.001
**NH, n(%)**	23(65.7%)	7(63.6%)	1(25.0%)	11(91.7%)	1(0.9%)	<0.001

* Median (IQR)

** Kruskal-Wallis test

CB= Chromosomal bridge, MPM= Multipolar mitosis, MN= Micronucleus, NB= Nuclear bridges, CS-chromatin string, NH=Nuclear heterogeneity, CI=chromosomal instability, n-numbers of sample

There was a significant difference in all CI morphology markers between B-ALL, AML, relapse, and MDS compared to patient remission samples. However, there was no significant difference in the CI markers between B-ALL, their relapse samples, and AML with the Dunn test.

All five patient groups were further sub-grouped into three: 1—patients with active disease (including B/T-ALL, AML, and MDS); 2—patients with disease in relapse; and 3—patients with disease in remission (remission group). Table 3 shows the scoring of the morphology markers of CI in these three groups.

**Table 3:** Morphological markers of CI in patients with active disease, relapse disease and disease in remission of acute leukaemia and MDS (n=178 sample) at University College of Medical Sciences and GTB Hospital, Delhi, North India, between June 2019 and June 2021

Parameter (CI markers) (IQR)	Patients with active disease (Group1)	Patients with Relapse disease (Group2)	Patients with disease in remission (Group 3)	P-value**
MN*	3(2, 6)	3 (1.5, 5.5)	0(0, 0)	<0.001
NB*	20(9, 30)	31.5 (21.5, 38.5)	1(0, 2)	<0.001
MPM*	2(1, 5)	5 (3, 6)	0(0, 0)	<0.001
CB*	0(0, 1)	0 (0, 0.5)	0(0, 0)	<0.001
Laggards*	0(0, 1)	0 (0, 1)	0(0, 0)	<0.001
CS*	6(2, 12)	10 (7, 12)	0(0, 0)	<0.001
**NH, n (%)**	31(62.0%)	11 (91.7%)	1(0.9%)	<0.001

* Median (IQR)

** Kruskal-Wallis test

CB= Chromosomal bridge, MPM= Multipolar mitosis, MN= Micronucleus, NB= Nuclear bridges, CS-chromatin string, NH=Nuclear heterogeneity, CI=chromosomal instability. MDS-Myelodysplastic syndrome

All the CI morphology markers (Micronucleus, NB, MPM, CB, CS, and Laggards) were significantly higher in patients with active disease and disease with relapse than those in remission. This suggests that the CI morphology markers are strongly associated with active disease of haematological malignancies and relapsed disease (Table 3).

Using the Dunn test, all morphology markers (MN, NB, MPM, CB, and NH) were significantly high in active disease (ALL, AML, MDS) vs. remission (P .00001) and relapse vs. remission (p= .0001 to .0177). However, there was no significant difference (P-value=0.30) between the active disease group and the relapse group of disease for all morphology markers of CI.

### Comparison of survivors and non-survivors

**A: Clinical features (Table 4):** The fever, bone pain, weight loss, loss of appetite, fatigue/weakness, splenomegaly, hepatomegaly, and lymphadenopathy were significantly more among non-survivors than survivors, whereas anaemia was not different among both groups.

**Table 4:** Clinical manifestations between non-survivors and survivors among acute leukaemia and MDS patients in GTB Hospital, Delhi, North India, between June 2019 to June 2021

Clinical features	Non-survivors n(%)	Survivors n(%)	P-value*
**Fever**	26(37.14%)	22(20.37%)	**0.014**
**Bone pain**	18(25.71%)	13(12.04%)	**0.019**
**Weight loss**	31(44.29%)	31(28.70%)	**0.033**
**Loss of appetite**	33(47.14%)	30(27.78%)	**0.008**
**Fatigue/weakness**	37(52.86%)	38(35.19%)	**0.020**
**Paleness/anaemia**	60(85.71%)	86(79.63%)	0.302
**Hepatomegaly**	18(25.71%)	12(11.19%)	**0.011**
**Splenomegaly**	18(25.71%)	11(10.60%)	**0.006**
**Lymphadenopathy**	13(18.57%)	9(8.33%)	**0.043**

* Chi-square test.

All CI markers were significantly increased among nonsurvivors compared to survivors (Table 5).

**Table 5:** Morphological markers of chromosomal instability in non-survivors ((n=38) and survivors (n=63) among acute leukaemia and MDS patients

CI marker	Non-survivors	Survivors	P-value**
MN*	1(0, 4)	0(0, 1)	<0.001
NB*	6.5(1, 21)	1.5(0, 4)	<0.001
MPM*	1(0, 4)	0(0, 1)	<0.001
CB*	0(0,0)	0(0,0)	0.008
Laggards*	0(0, 1)	0(0, 0)	0.003
CS*	1(0, 8)	0(0, 1)	<0.001
**NH, n (%)**	26(37.2%)	17(15.8%)	<0.001

* Median (IQR)

** Mann-Whitney test for all except NH (Chisquare test)

CB= Chromosomal bridge, MPM= Multipolar mitosis, MN= Micronucleus, NB= Nuclear bridges, NH=Nuclear heterogeneity, CI=chromosomal instability, CS-chromatin string

We also compared the CI markers among Survivors and Non-survivors for ALL MPM (p=0.048) and NH (p=0.023) with significantly higher in Non-survivors (Table S1) and CI markers in AML (Table S2). Values were higher in Non-survivors, though it didn't reach statistical significance, possibly due to the low number of samples in each category. After regression analysis, the CS and NH were significantly associated with the nonsurvivors group (p=0.039 and p=0.032) and Coefficient (95% Confidence interval) -.142 (-.279 to -.004) and -2.93 (-4.40 to -1.47), respectively.

### Comparison of responder and non-responder to therapy

The responder group (complete and partial response to therapy) and non-responder group (not responding to therapy and relapse cases) (a total of 164 samples included) were compared in (Table 6).

**Table 6:** Morphological markers of chromosomal instability in non-responder (n=72 samples) and responder (n=92 samples) to therapy among acute leukaemia and MDS patients

CI marker	Non-responder	Responder	P-value**
MN*	1(0, 3)	0(0, 1)	<0.001
NB*	3(1, 21)	2(0, 4)	<0.013
MPM*	1(0, 3)	0(0, 1)	<0.034
CB*	0(0,0)	0(0,0)	<0.013
Laggards*	0(0, 0)	0(0, 0)	<.0040
CS*	1(0, 6)	0(0, 1)	<0.031
**NH, n (%)**	24(66.7%)	12(33.3%)	<0.002

* Median (IQR)

** Mann-Whitney test for all except NH (Chisquare test)

CB= Chromosomal bridge, MPM= Multipolar mitosis, MN= Micronucleus, NB= Nuclear bridges, NH=Nuclear heterogeneity, CI=chromosomal instability, CS-chromatin string

All CI markers of morphology are significantly higher in the non-responder group than in the responder group. On logistic regression analysis, the p-value with Coefficient (95% confidence interval) of CS (p=0.011) 0.224 (.05 to .397) and NH (p=0.010), .224 (0.05 to 0.397) were significantly associated with the non-responder group. The p-value and Coefficient (95% confidence interval) for other parameters are shown in (Table S4).

## Discussion

Our findings indicate that morphological markers of CI significantly increased in acute leukaemia, MDS, and relapse cases compared to remission. Further, the CI markers were significantly higher among non-survivors compared to survivors. Interestingly, in the pilot study, Laggards did not considerably increase in AL and MDS and did not show a significant association with clinical manifestations.^8^ None of The CI markers increased among the Non-survivors in the previous study.^8^ This might be related to a small sample size. All CI markers were significantly increased in non-survivor groups, whereas in the previous study, only MPM, Laggards and MN increased significantly in the dead group.^8^ In the present study, we found that most clinical manifestations (fever, bone pain, weight loss, loss of appetite, fatigue, hepatomegaly, splenomegaly, and lymphadenopathy) were considerably higher in non-survivors than survivors. All morphological markers were significantly higher in the non-responder group than in the responder group in the present study. These findings suggest that CI markers of morphology could be alternative diagnostic and prognostic biomarkers for acute leukaemia and MDS compared to cytogenetics. These biomarkers (CI markers of morphology) are easy to perform and interpret in poor funding institutes or resource-poor countries lacking in high-cost equipment.

Regression analysis for non-survivor vs survivors and non-responder vs responder groups and morphology markers of CI, chromatin string and nuclear heterogenicity were significantly higher in non-survivors and nonresponder groups of patients. That indicates chromatin string and nuclear heterogenicity are independently associated with the patient's poor outcomes or not associated with other factors. This indicates that chromatin string and nuclear heterogenicity are independently poor prognostic factors in acute leukaemia.

Few studies on the morphology markers of CI in solid tumours like breast and pancreatic cancers are described in the literature.^9^,^10^,^11^,^12^ A previous study by Narin et al. (2012, Istanbul) reported that micronucleus was significantly (p<0.01) higher in patients with malignant melanoma than in healthy controls.^13^ In solid malignant breast tumours, CI markers such as micronucleus, multipolar mitosis, chromatin bridge and nuclear budding were higher than in benign lesions.^9^,^14^ In contrast to these studies, our study demonstrated multiple morphology markers of CI in haematological malignancies (AL and MDS). Our findings are corroborated by a few previous studies that reported significantly higher numbers of some morphology markers of CI, such as micronucleus in acute leukaemia.^15^,^16^ The present study described multiple morphological CI markers (micronucleus, MPM, CB, NB, CS, Laggards, and NH). Most of the CI markers significantly increased in AL and MDS compared to other studies.^13^,^15^,^16^ So, micronucleus and other morphology (multiple) markers can be used for the diagnosis and prognosis of AL and MDS. We found that all CI markers significantly increased active disease and relapse group of disease compared to remission groups of AL and MDS; none of the studies reported such a significant difference of all CI markers.^13^,^15^,^16^,^17^ Few studies on cytology smears were reported that significantly increased the number of CI markers of morphology in malignant effusion cytology smears compared to benign effusion cytology and higher grade of carcinoma breast.^18^,^19^,^20^ These findings corroborated our study results.

We found that all the CI markers also significantly increased in Non-survivors. However, after regression analysis, the chromatin string and nuclear heterogeneity correlated with the non-surviving group. Compared to other studies (one CI marker- micronucleus), we found that seven CI markers of morphology were significantly higher in the Non- survivor group than in the Survivors group.^13^,^15^,^16^,^17^ These CI markers represent the aggressiveness of diseases like acute leukaemia and MDS. When we compared ALL and AML for CI markers among survivors and non-survivors and found values were higher in non-survivors for all CI markers, MPM and NH significantly increased in non-survivor groups of ALL, but other markers didn't reach statistical significance (Table S1 and S2), which might be related to the low number of samples in each category. We did not find such a comparison in any literature. We also correlated morphology markers of CI and therapeutic response in MDS and AL. All CI markers significantly increased in non-responder groups than in responder groups. Similar results were reported by Wang et al. only with one CI marker micronucleus (low-frequency of micronucleus respond well than the high-frequency group).^16^

Some other studies also described the role of the micronucleus in the prognosis of acute leukaemia, and the results were like ours.^15^,^16^ Wang et al. demonstrated that acute leukaemia patients with low micronucleus frequencies had a significantly better response to therapy and better survival rates than those with high micronucleus frequencies.^16^ Lisboa et al. found that CI had adverse prognostic value in all types of acute myeloid leukaemia (de novo AML, secondary AML, and therapy-related AML) and reported that its roles might be either age-related or reflect the heterogeneity of the disease.^15^ Other studies have also demonstrated the role of MN in the poor prognosis of acute leukaemia.^15^,^16^ These studies described only one CI marker, whereas we reported multiple markers of CI related to prognosis.

The index study found fever, bone pain, weight loss, loss of appetite, fatigue, hepatomegaly, splenomegaly, and lymphadenopathy significantly higher in non-survivors (having higher scores of CI markers) than in the survivors group. Some studies also demonstrated such adverse reactions and correlated with CI markers.^13^,^16^ In our study, hepatomegaly and splenomegaly were significantly higher in the Non-survivor group than the Survivor's group, and most of the patients were in the pediatric age group in the study, which might be related to it. Few studies reported hepatomegaly positively correlated with age and subtypes of acute leukaemia 41.8% and 58.8% of pre-B and T-ALL patients.^21^,^22^

In solid tumours, morphology markers of chromosomal instability were correlated with tumour grading and tumour staging on cytology smears.^18^,^20^,^23^ The present study showed no significant difference in all CI markers between B-ALL and AML groups, consistent with other studies.^16^,^17^ We also did not find a significant difference in morphology markers of CI between the relapse group and acute leukaemia groups. Few studies have reported that MN appears ahead of chromosome aberration in peripheral blood lymphocytes, which predicts cancer risk.^24^,^25^

Chromosomal instability has been associated with intrinsic multi-drug resistance in solid tumours.^26^ Wang et al. reported that liver function, bone marrow toxicity, gastrointestinal toxicity, and ECG (electrocardiogram) changes were more evident in the patient group with high micronucleus frequency than in the group with low micronucleus frequency. Still, the difference was not statistically significant.^16^ Similarly, in this study, we found a non-responder group of patients with significantly higher CI markers than the responder to therapy. Heilig et al. reported elevated levels of CI in MDS patients associated with poor outcomes by FISH technique and other studies also poor prognosis related to an increased number of CI markers.^7^,^15^,^16^,^26^ Similarly, in our study, high levels of CI markers in non-survivors and non-responder groups of patients. A high level of CI markers is an indicator of poor prognosis. We recommended the incorporation of CI markers of morphology in the routine practice of reporting as Lisboa et al. recommended.^15^

Early diagnosis of acute leukaemia and MDS and identification of prognostic level (good and bad prognosisbased scoring of CI markers) will help in therapeutic implementation that may increase the overall quality of life and the survival of patients. CI markers of morphology can be used to diagnose and see the treatment response. Morphology markers of CI are easy to identify and more cost-effective than a karyotyping method because they do not require separate specimen preparations, such as karyotyping; they can be performed on routine examinations of BMA and biopsy. There is a lack of studies comparing the cytogenetics of CI markers with morphology markers of CI on BMA in AL, but few studies described chromosomal instability with Q- FISH and telomere shortening in papillary urothelial neoplasms and solid tumours of oesophagus.^27^-^28^

### Limitations

It is a retrospective study with a few missing data on follow-up and cytogenetics. The morphological markers of CI were not compared with cytogenetic techniques.

## Conclusion

Multiple morphological markers of CI (MN, NB, MPM, CB, Laggard, CS and NH) were significantly higher among patients with AL and MDS compared to remission cases of acute leukaemia and MDS. Furthermore, all the morphological markers of CI were higher in Non-survivors than in Survivors. Thus, we recommend incorporating morphological markers of CI into routine reporting systems to assist in prognostication before reports from sophisticated techniques are available because these markers are easy to identify and cost-effective.
